# Modulation of vagal activity may help reduce neurodevelopmental damage in the offspring of mothers with pre-eclampsia

**DOI:** 10.3389/fimmu.2023.1280334

**Published:** 2023-11-02

**Authors:** Eric Alonso Abarca-Castro, Ana Karen Talavera-Peña, José Javier Reyes-Lagos, Enrique Becerril-Villanueva, Gilberto Pérez-Sanchez, Francisco R. de la Peña, José Luis Maldonado-García, Lenin Pavón

**Affiliations:** ^1^ Departamento de Ciencias de la Salud, Universidad Autónoma Metropolitana-Lerma (UAM-L), Lerma, Mexico; ^2^ Facultad de Medicina, Universidad Autónoma del Estado de México (UAEMéx), Toluca de Lerdo, Mexico; ^3^ Laboratorio de Psicoinmunología, Dirección de Investigaciones en Neurociencias, Instituto Nacional de Psiquiatría Ramón de la Fuente Muñiz, Mexico City, Mexico; ^4^ Unidad de Fomento a la Investigación, Dirección de Servicios Clínicos, Instituto Nacional de Psiquiatría Ramón de la Fuente Muñiz, Mexico City, Mexico; ^5^ Departamento de Inmunología, Escuela Nacional de Ciencias Biológicas, Instituto Politécnico Nacional, Mexico City, Mexico

**Keywords:** maternal immune activation, pre-eclampsia, cholinergic anti-inflammatory pathway, neurodevelopmental outcomes, vagus nerve stimulation, quality of life

## Abstract

Maternal Immune Activation (MIA) has been linked to the pathogenesis of pre-eclampsia and adverse neurodevelopmental outcomes in the offspring, such as cognitive deficits, behavioral abnormalities, and mental disorders. Pre-eclampsia is associated with an activation of the immune system characterized by persistently elevated levels of proinflammatory cytokines, as well as a decrease in immunoregulatory factors. The Cholinergic Anti-inflammatory Pathway (CAP) may play a relevant role in regulating the maternal inflammatory response during pre-eclampsia and protecting the developing fetus from inflammation-induced damage. Dysregulation in the CAP has been associated with the clinical evolution of pre-eclampsia. Some studies suggest that therapeutic stimulation of this pathway may improve maternal and fetal outcomes in preclinical models of pre-eclampsia. Modulation of vagal activity influences the CAP, improving maternal hemodynamics, limiting the inflammatory response, and promoting the growth of new neurons, which enhances synaptic plasticity and improves fetal neurodevelopment. Therefore, we postulate that modulation of vagal activity may improve maternal and fetal outcomes in pre-eclampsia by targeting underlying immune dysregulation and promoting better fetal neurodevelopment. In this perspective, we explore the clinical and experimental evidence of electrical, pharmacological, physical, and biological stimulation mechanisms capable of inducing therapeutical CAP, which may be applied in pre-eclampsia to improve the mother’s and offspring’s quality of life.

## 1 Introduction

Pre-eclampsia is a condition characterized by hypertension, inflammation, and organ damage during pregnancy, and it has been associated with cognitive deficits, behavioral abnormalities, and neurodevelopmental issues in the offspring (1). Evidence indicates that the Cholinergic Anti-inflammatory Pathway (CAP) could significantly impact the development of the fetus and the newborn exposed to pre-eclampsia by functioning as a neuroimmunological network that facilitates internal monitoring. This pathway connects the central nervous system (CNS) through the vagus nerve, regulating inflammation in the body (2). Previous research indicates that the downregulation of α7 nicotinic acetylcholine receptor (α7nAChR) has a significant impact on the CAP to regulate systemic inflammation, particularly in cases of pre-eclampsia (3). However, this downregulation and its potential link to the onset of pre-eclampsia are areas still under investigation. Similarly, while there is some evidence to suggest that dysregulation of the CAP may contribute to the development of pre-eclampsia, further studies are needed to substantiate this claim (4).

This perspective article explores some vagal stimulation techniques that modulate the CAP and improve outcomes in preclinical pre-eclampsia models. Particularly, we explored the potential of modulating vagal activity, including the use of electrical Vagus Nerve Stimulation (VNS), to enhance maternal and fetal outcomes by targeting immune dysregulation and promoting fetal neurodevelopmental alterations caused by inflammation. This perspective article discusses the role of inflammatory response in triggering pre-eclampsia and its impact on neurodevelopment, emphasizing the increased risk of neurodevelopmental problems and mental disorders in the offspring. We position the modulation of vagal activity, including but not limited to VNS, as a promising, safe, and efficient therapeutic intervention, primarily backed by preclinical findings, warranting further exploration. This perspective proposes that modulating vagal activity using electrical, pharmacological, magnetic, and physical techniques, such as breathing and respiratory stimulation, might be potential approaches to address inflammatory dysregulation. It is hypothesized that such techniques could potentially improve outcomes in pre-eclampsia, minimize neurodevelopmental damage, and enhance the quality of life for affected mothers and their offspring (a visual abstract is available as **Supplementary Material**).

## 2 The maternal immune system in pregnancy: implications for pre-eclampsia

During pregnancy, the maternal immune system undergoes essential adaptations to protect both the mother and fetus from antigenic challenges while maintaining tolerance to the fetal allograft (5). Evidence has described an association between the balance of pro- and anti-inflammatory cytokines and placental development and function (6). Some research suggests that an inflammatory response during pregnancy is associated with complications like pre-eclampsia (7, 8). In animal models of pre-eclampsia, it has been found an increase in serum concentration of IFN-γ, IL-6, TNF-α, and IL-17, in addition to a decrease in IL-4 and IL-10, accompanied by placental damage (9–12). Meanwhile, in women with pre-eclampsia, it has been found an increase in pro-inflammatory cytokine serum levels such as IL-2, IL-4, IL-6, IL-15, IL-16, IL-17, IL-22, IL-35, IFN-γ, and TNF-α, as well as a decrease in IL-10 concentration (13–19). Therefore, the IL-2/IL-10 and TNF-α/IL-10 ratios have been proposed to assess the risk of developing pre-eclampsia (20). IL-22/CCL22 and IL-2/IL-4 ratios have also been considered (19). Conversely, some other authors (18, 21) have reported no changes in pro-inflammatory cytokine levels in pre-eclampsia. Consequently, it has been proposed that differences in the inflammatory profile reported in the studies could be explained by the sample size, the polymorphisms found in the population, and the sample collection conditions (22). Present findings predominantly point towards an association between inflammation and pre-eclampsia, rather than a conclusive cause-and-effect dynamic. This accentuates the need for more in-depth investigations to elucidate the relationship between cytokines and pre-eclampsia.

Furthermore, growth restriction has been observed in animal models of pre-eclampsia (23, 24). Similarly, pre-eclampsia induces fetal growth restriction in humans, which has been considered a predictor of maternal and neonatal prognoses (25, 26). Additionally, prenatal exposure to pre-eclampsia has been identified as a risk factor for developing type 2 diabetes and cardiovascular disease (27). Also, in recent years the study of neurodevelopmental repercussions following prenatal exposure to pre-eclampsia has gained interest and has been associated with inflammatory alterations (12, 28).

It has been described that the presence of pro-inflammatory cytokines during pregnancy due to Maternal Immune Activation (MIA) affects neurodevelopmental processes in the fetus (29). IL-6 and IL-17 have been highly implicated in causing the priming of microglia and leukocytes, which increases the risk of developing neuropsychiatric diseases characterized by inflammatory cytokines (30–33).

IL-6 has been implicated in altered synaptogenesis in animal models and impaired functional connectivity in human frontoparietal networks (8, 34, 35). Conversely, IL-17 and TNF-α have been implicated in increased Blood-Brain Barrier (BBB) permeability and damage to the cerebral vasculature, as well as in neural tube defects (36–38). Finally, increased IFN-γ has been associated with white matter damage in the central nervous system in preterm neonates (39).

## 3 Maternal immune activation and the cholinergic anti-inflammatory pathway in pre-eclampsia

MIA is a term that refers to an increase in proinflammatory markers during pregnancy, it is characterized by the activation of the maternal immune system in response to infectious or infectious-like stimuli (40). This activation leads to a series of changes in cytokine levels and immune responses that can affect the developing fetus, particularly the central nervous system, and give rise to adverse phenotypes (41). MIA typically occurs during the middle to late stages of gestation which are considered a critical period for brain development. During this time, adverse environmental conditions can impact neurogenesis (42). Thus, MIA can impact fetal development and have long-term effects on the offspring, leading to various neurological disorders. According to relevant findings, these disorders share genes and molecular mechanisms and are potentially associated with the abnormal structure and dysregulation of the amygdala (43). Furthermore, evidence has shown a link between MIA and increased emotional and behavioral problems in offspring throughout their childhood and adolescence. The findings support the idea that MIA can pose a risk to children’s mental and neurodevelopmental health during prenatal programming (44).

Several pregnancy complications, such as preterm birth, fetal growth restriction, and pre-eclampsia, may involve MIA. Pre-eclampsia is mainly linked to neurodevelopmental problems such as Autism Spectrum Disorder (ASD) and neurodevelopmental delay (45). However, it is possible that the inflammatory immune response seen in preeclamptic women has similar consequences on microglia, as observed in MIA, and may affect fetal microglia stability (45). A recent preclinical study revealed that fetal brain inflammation might be linked to the pathological mechanism connecting maternal pre-eclampsia and brain dysfunction in offspring. These findings are intriguing, as they suggest that pre-eclampsia in mothers might lead to altered inflammatory conditions in the fetal brain (46).

CAP inhibits the release of pro-inflammatory cytokines. This process requires the activation of the vagus nerve through α7 receptors (47). Tracey’s research has highlighted the vagally mediated CAP as an effective means of rapidly reducing inflammation (48). Relevant preclinical findings suggest that the activation of the α7nAChR attenuates pre-eclampsia-like symptoms, and this protective effect is likely the result of inhibiting inflammation through the NF-κB p65 pathway (49). According to a pertinent review by Wedn et al., the involvement of both peripheral and central cholinergic pathways plays a crucial role in the development and progression of pre-eclampsia. Interestingly, increasing the CAP could be a potential strategy for effectively managing pre-eclampsia and protecting against its associated maternal and fetal complications (50).

## 4 The impact of pre-eclampsia on neurodevelopment: insights into cognitive, behavioral, and mental disorders in offspring

Neurodevelopmental disorders are an issue that, according to research, may be related to insults during maternal pregnancy (51). Therefore, maternal health care during this period is crucial, as it is vital for both the mother's and the offspring's health (52). The inflammatory process and MIA caused by pre-eclampsia in the mother can affect both the mother's well-being and potentially lead to short, medium, and long-term problems in the neurodevelopment of the offspring (1, 53, 54). This association can generate an impact on the neurological basis of the offspring in the acquisition, retention, or application of specific skills or sets of information, as well as in memory, perception, language, problem-solving, executive function control, or social interaction; which produces a deficit in personal, social, academic, or occupational development (55). Several reports have found a significant association between pre-eclampsia and an increased risk of neurodevelopmental problems in offspring and mental disorders (56). In a recent study, researchers found that children born to mothers with pre-eclampsia were at higher risk of developmental delays, including cognitive, motor, and language difficulties (57), an increased risk of loss of cognitive functioning (58–60), Attention Deficit-Hyperactivity Disorder or ADHD (1, 28, 60–62), (1, 60, 61, 63), schizophrenia (28, 61), and epilepsy (1, 60, 61).

The aforementioned clinical conditions may be associated with functional and neurochemical changes described in the offspring of mothers with pre-eclampsia, such as altered dopamine levels, a neurotransmitter involved in learning and reward in specific brain regions (58, 64, 65), and the regulation of oxidative stress in the developing brain (61). The underlying mechanisms of this association are not yet fully understood. However, it is thought to involve disrupting normal fetal brain development due to impaired blood flow and oxygen supply. Preclinical studies have shown that pre-eclampsia can cause changes in the fetal brain’s structure and function, leading to altered behavior and cognitive deficits (4).

On the other hand, cardiovascular and metabolic diseases have been associated with the late-life outcomes of preeclamptic offspring (66). Furthermore, some neurodevelopmental and psychiatric disorders, such as ADHD and disruptive behavioral disorders, were significantly observed as early as six years after birth in the offspring of mothers with pre-eclampsia and perinatal complications in Finland (67). These findings have been reported in Taiwan, where not only ADHD was significantly increased, but also ASD and intellectual disability (60). Also, these psychiatric disorders cause significant distress in parents and children, resulting in disproportionate expenses during the assessment and treatment of these conditions. Further, the economic impact of these situations can be seen in the financial dependence of young adults with childhood ADHD (68).

## 5 Modulation of vagal nerve activity and potential applications in pregnancy

Electrical Vagus Nerve Stimulation (VNS) is a contemporary method that applies electrical pulses to the vagus nerve. This affects both upward- and downward-projecting nerve fibers, influencing the brainstem and internal organs. It plays a role in modulating autonomic functions as well as neuroendocrine and neuroimmunological systems (69). Vagal efferent fibers, which are prevalent in internal organs, facilitate communication between the nervous and immune systems, primarily via the CAP. The VNS has been used for over 20 years, but there is limited information on its safety during pregnancy. Voinescuo and Meador analyzed data from the International Registry of Antiepileptic Drugs and Pregnancy (EURAP) to assess pregnancy outcomes in 26 pregnancies among 25 women with epilepsy who used implanted VNS device during pregnancy. The sample size was too small to draw firm conclusions on the safety of implanted vagus nerve stimulators in pregnancy, but the study adds to the literature and encourages further research to improve the evidence for managing women with epilepsy during pregnancy (70). Preclinical relevant evidence has studied the effect of VNS during pregnancy and its underlying mechanisms in pre-eclampsia. Pregnant rats were used as a model, and implanted VNS therapy was found to decrease systolic blood pressure and urinary protein levels while mitigating abnormal pregnancy outcomes. VNS also reduced the inflammatory response by decreasing cytokine levels in the maternal serum, increasing the expression of placental α7nAChR and effectively inhibited placental NF-κB p65 activation. The study suggests that maternal VNS treatment is safe and protective in a pregnant rat model (71).

Transcutaneous auricular vagus nerve stimulation (taVNS), a non-invasive alternative to implanted VNS, has garnered substantial research interest due to its comparable benefits (72). Recognized as a potentially safe and feasible treatment, it is imperative for upcoming research on taVNS to meticulously evaluate any adverse events (73). Moreover, there is evidence indicating that taVNS can amplify cardiac vagal activity, reflected by heart rate variability (HRV) (74). Consequently, we speculate that, unlike the invasive implanted VNS, taVNS might serve as a pivotal tool in future studies on preeclamptic women to potentially alleviate their symptoms and possibly mitigate neurodevelopmental damage in their offspring.

In addition to the electrical VNS, other molecules may stimulate the CAP, such as extracellular monomeric ubiquitin (mUB) or pyridostigmine, a carbamate inhibitor of acetylcholinesterase whose administration can increase the concentration of circulating acetylcholine, presents an extensive range of safety in recommended doses (30-60 mg every 4-8 h) since the drug does not cross the placenta in significant amounts (75). However, its consumption may induce mainly intestinal side effects in patients (76). The clinical relevance of increasing the circulating cholinergic tone in an individual is that a high vagal tone favors the decreased serum levels of inflammatory molecules such as TNF-α, as well as cortisol and epinephrine (76), a very significant positive fact of this therapeutic strategy was observed when pyridostigmine was administered in patients affected by COVID-19 in which its administration increases the survival levels in patients (77). An even more novel option is the use of mUB, which is a small (8.6 kDa) and heat-stable protein higly-conserved in all eukaryotic cells (78). A large body of evidence shows *in vitro* and *in vivo* that pure mUB administration induces immunomodulatory effects, including decreasing macrophage and lymphocyte cellular functions, such as chemotaxis, proliferation, and cytokine secretion (78, 79). It is suggested that these immunomodulatory effects induced by mUB result from partial blockade of the CXCR4/CXCL12 axis. CXCR4 is constitutively expressed in the membrane of leukocytes in monomeric or dimeric form; it is associated with G proteins α and βγ subunits, β-arrestin, and JAK/STAT, and its activation favors chemotaxis, cell proliferation, and other cell activation phenomena (78).

mUB has been described as the main component of a Dialyzable Leukocyte Extract (DLE) (71). Interestingly, this complex drug induces a decrease in circulating levels of proinflammatory cytokines (TNF-α, IL-6), cortisol, and catecholamines when orally administered in major depression patients (72) as well as in murine (73) and canine infectious models (74, 75). Interestingly, the DLE and mUB alone increase the percent of survival of HSV-1-infected mice (80). This body of evidence suggests that the biological effects of the DLE in infectious models could be partially attributed to mUB, its main component.

Finally, the utilization of relaxation methods involves various mind-body techniques, including breathing training, progressive relaxation, and guided imagery, as supported by Geranmayeh et al. (81). Deep breathing-based yoga, a commonly practiced approach (82), has shown evidence of stimulating the vagus nerve, activating the parasympathetic nervous system and enhancing HRV. Moreover, the Food and Drug Administration (FDA) has approved magnetic stimulation of the vagus nerve to treat epilepsy and depression (83), demonstrating the potential of modulating vagal nerve activity through safe and established practices.

## 6 Perspective

**Figure 1:**
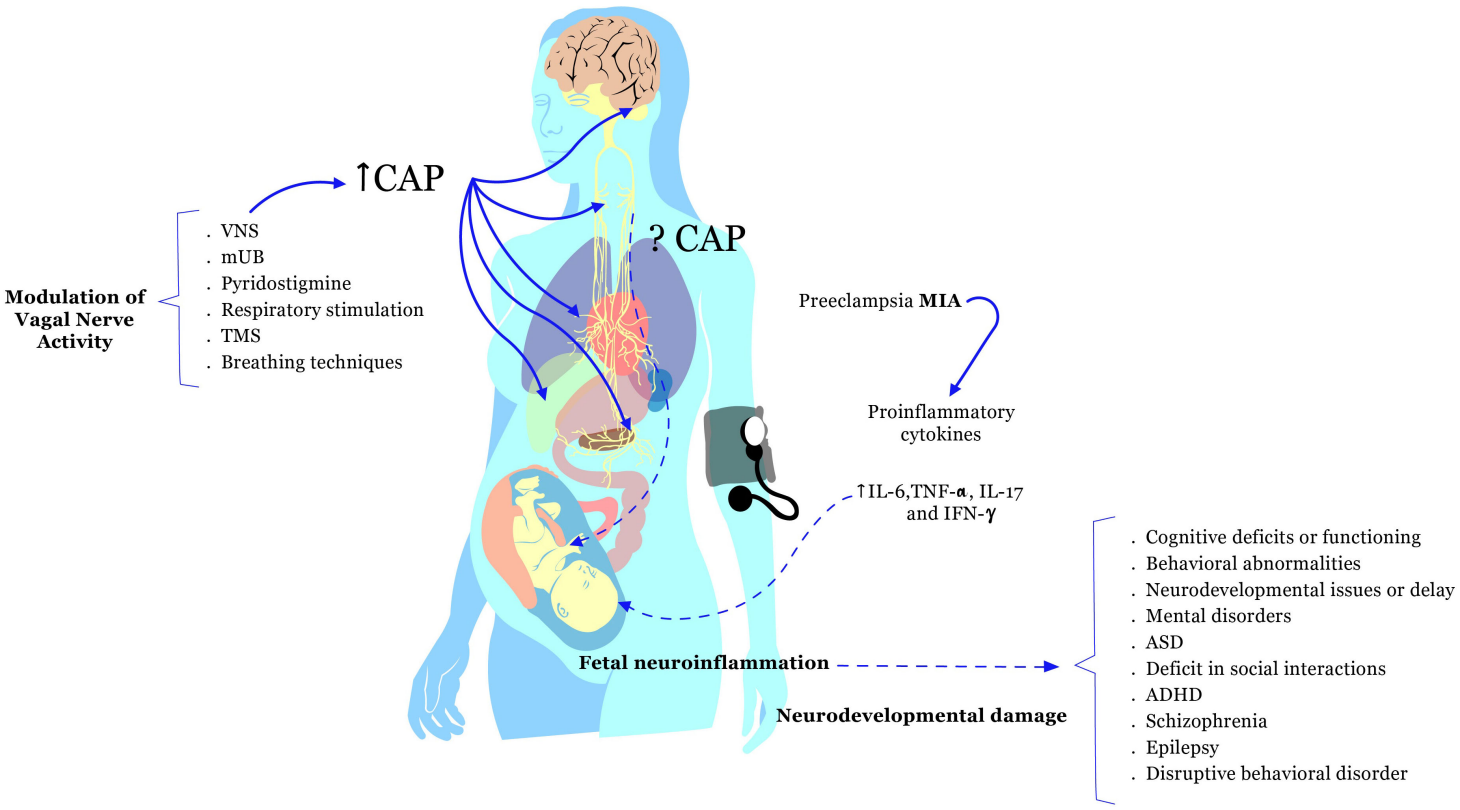
The inflammatory response during pregnancy can initiate complications, such as pre-eclampsia. Elevated pro-inflammatory cytokines, including interleukin-6 (IL-6), IL-17, tumor necrosis factor-alpha (TNF-α), and interferon-gamma (IFN-γ), are linked to this phenomenon. These pro-inflammatory cytokines may primarily mediate Maternal Immune Activation (MIA) during pregnancy and could impact fetal neurodevelopmental processes. The pathogenesis of pre-eclampsia might involve the disruption of the Cholinergic Anti-inflammatory Pathway (CAP) and fetal neuroinflammation. Pre-eclampsia has been associated with adverse neurodevelopmental outcomes in the offspring, such as cognitive deficits, behavioral abnormalities, mental disorders, neurodevelopmental issues, Autism Spectrum Disorder (ASD), Attention Deficit-Hyperactivity Disorder (ADHD), and others. Potential strategies to enhance maternal and fetal outcomes in pre-eclampsia involve modulating vagal nerve activity. This modulation can be achieved through techniques like Electrical Vagus Nerve Stimulation (VNS), pharmacological interventions (oral extracellular monomeric ubiquitin or mUB, and pyridostigmine), physical approaches (respiratory stimulation and breathing techniques), and Transcranial Magnetic Stimulation (TMS) in preeclamptic pregnant women.

Based on insights garnered from various studies, we propose that the modulation of vagal activity might offer a beneficial avenue for improving maternal and fetal outcomes in cases of pre-eclampsia. Pre-eclampsia, known for its links to unfavorable neurodevelopmental results in offspring, stems from a potential dysregulation within the Cholinergic Anti-inflammatory Pathway (CAP) and Maternal Immune Activation (MIA). There have been encouraging observations of reduced inflammation through electrical (VNS), physical, magnetic, and pharmacological methodologies that modulate the CAP. Such interventions may potentially improve maternal hemodynamics and bolster fetal neurodevelopment (as seen in **Table 1** and **Figure 1**). Furthermore, exploring other molecular agents, such as extracellular monomeric ubiquitin (mUB) or pyridostigmine, to modulate the CAP seems promising. These strategies might address immune system imbalances, thus enhancing the quality of life for mothers dealing with pre-eclampsia. As a result, these experimental insights pave the way for the proposition that stimulating the CAP in pre-eclampsia offers a novel therapeutic tactic to mitigate the neurodevelopmental challenges posed by the condition. However, it is imperative to emphasize the necessity for additional research, particularly those studies backed by robust scientific evidence. This is crucial to ascertain the efficacy and safety of techniques to modulate vagal activity, especially when considering its application in pregnant women. As with any potential therapeutic approach, the weight of the evidence will guide its widespread acceptance and application in the clinical realm.

**Table 1:** Modulation of vagal nerve activity through electrical, pharmacological, physical, and magnetic stimulation.

Treatment	Mechanism of action	Dose	Safety
**Electrical Vagus Nerve Stimulation (VNS)**	Initially associated with increased extracellular norepinephrine in epilepsy, VNS has been found to raise free GABA levels in the cerebrospinal fluid (84)	0.75-3.5 mA (85, 86)	Implanted vagus nerve stimulator shows a low risk of fetal malformation and does not affect downstream target organs. Favorable evidence outweighs adverse evidence, suggesting that implanted VNS devices may be safe during pregnancy (86)
**Oral extracellular monomeric ubiquitin (mUB)**	Mild agonist of the CXCR4 receptor expressed on the vagal nerve in the stomach (87)	1.003 µg/dose (80)	Is a self-protein with no primary toxicity, and there are no reports of toxic effects from its consumption
**Pyridostigmine**	A carbamate inhibitor of acetylcholinesterase (75)	30-60 mg every 4-8 h (75)	The drug does not cross the placenta in significant amounts (75)
**Respiratory stimulation**	Increased vagal tone is linked to slow, deep breathing with higher tidal volume, whereas irregular, shallow, and fast breathing is associated with increased sympathetic tone (82)	At least 6 hours of continuous positive airway pressure (CPAP) (88)	CPAP use during pregnancy may lower blood pressure and reduce the risk of pre-eclampsia. No severe side effects or complications were reported in any study (89)
**Transcranial magnetic stimulation (TMS)**	TMS activates descending fibers, causing electrical impulses to travel through the spinal cord and to the peripheral nerve, resulting in muscle twitching (83)	5 to 25 Hz (90)	It is safe and well-tolerated, making it a potential additional treatment for depression in pregnant patients. There is no evidence suggesting that TMS poses a risk to the fetus when administered to the mother during pregnancy (91)
**Breathing techniques**	It increases endorphin hormone secretion, reduces adrenaline and cortisol levels, and lowers heart rate and blood pressure in pregnant women (81)	Jacobson’s progressive muscle relaxation technique was used twice a week for six weeks. Sessions lasted about 45 minutes (92)	Breathing techniques and relaxation are safe and harmless complementary treatments during pregnancy (81)

## Author contributions

EA-C: Conceptualization, Investigation, Resources, Writing – original draft, Writing – review & editing. AT-P: Conceptualization, Investigation, Resources, Writing – original draft, Writing – review & editing. JR-L: Resources, Writing – original draft, Writing – review & editing. EB-V: Resources, Writing – original draft, Writing – review & editing. GP-S: Resources, Writing – original draft, Writing – review & editing. FD-O: Resources, Writing – original draft, Writing – review & editing. JM-G: Resources, Writing – original draft, Writing – review & editing. LP: Conceptualization, Investigation, Resources, Writing – original draft, Writing – review & editing.
