# Supplementary material for: Modulation of vagal activity may help reduce neurodevelopmental damage in the offspring of mothers with pre-eclampsia
Source: Front Immunol. 2023 Nov 2;14:1280334. doi: 10.3389/fimmu.2023.1280334 (PMC10653300; doi:10.3389/fimmu.2023.1280334)

MODULATION OF VAGAL ACTIVITY MAY HELP REDUCE NEURODEVELOPMENTAL DAMAGE IN THE OFFSPRING OF MOTHERS WITH PRE-ECLAMPSIA

Background & Maternal Immune System

Understanding the role of the maternal immune system in pre-eclampsia.

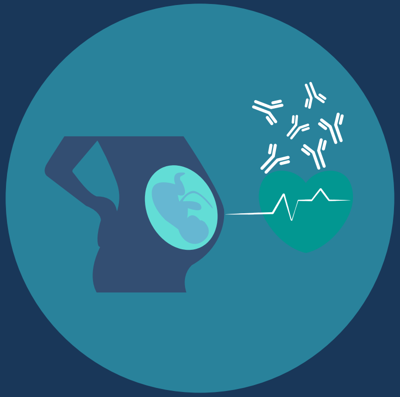

Neurodevelopmental Impact & Vagal Modulation

Neurodevelopmental implications for offspring. Vagal modulation (through Electrical, Pharmacological, Physical, and Magnetic Stimulation) as a potential solution.

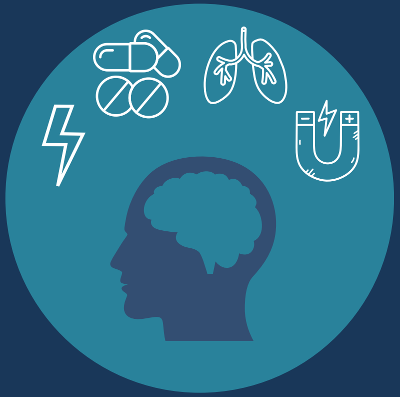

Perspective & Outcomes

Modulation of vagal activity may help reduce neurodevelopmental damage in the offspring of mothers with pre-eclampsia.

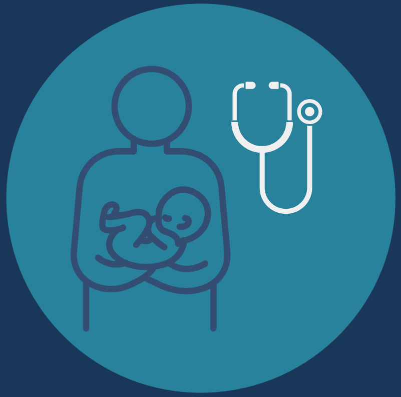

Supplement: Supplementary file 1 [file Image_1.pdf]
